# Cu^2+^-Ion-Substitution-Driven Microstructure and Microwave Dielectric Properties of Mg_1−*x*_Cu*_x_*Al_2_O_4_ Ceramics

**DOI:** 10.3390/nano12193332

**Published:** 2022-09-24

**Authors:** Yuanming Lai, Ming Yin, Baoyang Li, Xizhi Yang, Weiping Gong, Fan Yang, Qin Zhang, Fanshuo Wang, Chongsheng Wu, Haijian Li

**Affiliations:** 1 School of Mechanical and Electrical Engineering, Chengdu University of Technology, Chengdu 610059, China; 2 Guangdong Provincial Key Laboratory of Electronic Functional Materials and Devices, Huizhou University, Huizhou 516001, China; 3 State Key Laboratory of Electronic Thin Films and Integrated Devices, University of Electronic Science and Technology of China, Chengdu 610054, China; 4 Science and Technology on Combustion and Explosion Laboratory, Xi’an Modern Chemistry Research Institute, Xi’an 710065, China

**Keywords:** microwave dielectric properties, MgAl_2_O_4_ ceramics, solid solution, lattice energy, bond energy

## Abstract

In this work, Cu-substituted MgAl_2_O_4_ ceramics were prepared via solid-state reaction. The crystal structure, cation distribution, and microwave dielectric properties of Mg_1−*x*_Cu*_x_*Al_2_O_4_ ceramics were investigated. Cu^2+^ entered the MgAl_2_O_4_ lattice and formed a spinel structure. The substitution of Cu^2+^ ions for Mg^2+^ ions contributed to Al^3+^ ions preferential occupation of the octahedron and changed the degree of inversion. The quality factor (Qf) value, which is correlated with the degree of inversion, increased to a maximum value at *x* = 0.04 and then decreased. Ionic polarizability and relative density affected the dielectric constant (ε_r_) value. The temperature coefficient of the resonant frequency (τ_f_) value, which was dominated by the total bond energy, generally shifted to the positive direction. Satisfactory microwave dielectric properties were achieved in *x* = 0.04 and sintered at 1550 °C: ε_r_ = 8.28, Qf = 72,800 GHz, and τ_f_ = −59 ppm/°C. The Mg_1−*x*_Cu*_x_*Al_2_O_4_ solid solution, possessing good performance, has potential for application in the field of modern telecommunication technology.

## 1. Introduction

The microwave dielectric ceramics have been extensively applied in various fields, including fifth-generation wireless systems, intelligent transmission systems, and ultrahigh-speed wireless local area networks [1,2,3]. In the application of millimeter waves, there is an urgent requirement for microwave dielectric ceramics with excellent performance in the following areas: a high quality factor (Qf) to enable microwave frequency selectivity, a low dielectric constant (ε_r_) to shorten the delay time of signal propagation, and a near-zero temperature coefficient of resonant frequency (τ_f_) to ensure the stability of frequency against temperature changes [4,5]. However, few single-phase materials can meet these requirements simultaneously due to the mutual restrictions of the three parameters. In general, ideal ε_r_ and Qf values can be obtained by selecting material systems, whereas the near-zero τ_f_ value is tailored through two materials with opposite τ_f_ values [6,7,8,9]. However, this approach tends to deteriorate the ε_r_ and Qf values.

Previous studies have found that the τ_f_ value is related to octahedral distortion in some ceramic crystals [10,11]. Microwave dielectric ceramics with superior τ_f_ values can be obtained by adjusting the distortion of the octahedron without deteriorating the ε_r_ and Qf values [4]. Therefore, it is important to improve the microwave dielectric properties utilizing the crystal structure. In general, the spinel structural degrees of freedom, such as the cell parameters, the oxygen fractional coordinates, and degrees of inversion, can be tailored via substitution [12]. MgAl_2_O_4_ is known to have a typical cubic spinel belonging to symmetry group Fd-3m (227); the molecular formula is [Mg_1−λ_Al_λ_]^IV^[Al_2−λ_Mg_λ_]^VI^O_4_, where λ value, which is related to the degree of inversion [13,14], represents the occupation of Al^3+^ cations at tetrahedral site.

MgAl_2_O_4_ ceramic, which generally exhibits a Qf value of ~68,900 GHz (the highest Qf value is over 200,000 [15]) and a low ε_r_ value (~8.75), is one of the candidate material for a millimeter-wave communication substrate [16]. However, it has a large negative τ_f_ value (~−75 ppm/°C). It has been reported that MgAl_2_O_4_-based composite dielectric ceramics, such as MgAl_2_O_4_-TiO_2_ and MgAl_2_O_4_-(Ca_0.8_Sr_0.2_)TiO_3_, have near-zero τ_f_ values [6,7]. However, the ε_r_ and Qf values are also deteriorated in this system. It is worth mentioning that the τ_f_ value in MgAl_2_O_4_ can be improved through the crystal structure [17,18]. Previously, in MgAl_2_O_4_ ceramics, it has been shown that the enhancement in Qf value corresponds to the cation distribution [19]. Moreover, the degree of inversion in MgAl_2_O_4_ ceramics, prepared by the solid-state reaction or molten-salt reaction routes, has also been investigated [15]. A high degree of inversion represents a high Qf value, and the preferential occupation of Al^3+^ could enhance the covalency of M-O bonds in a [MO_4_] tetrahedron of MgAl_2_O_4_ (M = Mg and Al). Consequently, the cation distribution of Al^3+^ in MgAl_2_O_4_ can be discussed to ameliorate the microwave dielectric properties.

In general, the ionic radius of Cu^2+^ ion is close to that of Mg^2+^ ions [4,20,21], which is beneficial for forming Mg_1−*x*_Cu*_x_*Al_2_O_4_ solid solutions. Additionally, a significant Jahn–Teller effect can be observed when Cu^2+^ ions occupy the octahedral site in a spinel structure [22]. This can contribute to the regulation of the microstructure of MgAl_2_O_4_. In addition, CuO can also reduce the sintering temperature of ceramics [4,21]. Therefore, in this work, the Cu^2+^ ion was considered as a substitution of the Mg^2+^ ion in MgAl_2_O_4_. Mg_1−*x*_Cu*_x_*Al_2_O_4_ ceramics were synthesized through a solid-state route. The phase composition, microstructure, and microwave dielectric properties were investigated in detail.

## 2. Experimental Procedure

Mg_1−*x*_Cu*_x_*Al_2_O_4_ (*x* = 0, 0.04, 0.08, 0.12, 0.16, and 0.20) ceramics were synthesized via the conventional solid-state route. Analytic-grade purity MgO, CuO, and Al_2_O_3_ powders (Shanghai Macklin Biochemical Co., Ltd., Shanghai, China) with the range of particle size at 45–80 μm were used as starting materials, which were weighed and wet-mixed in deionized water using zirconia balls in a plastic container at 300 rpm for 4 h. The obtained slurries were dried and calcined in alumina crucibles at 1450 °C for 4 h. Subsequently, the calcined powders were ground into a fine form and pressed under a uniaxial pressure of 10 MPa into cylindrical disks with 12 mm diameter and 5–6 mm height. Samples were sintered at temperature levels ranging from 1450 to 1600 °C for 4 h.

To confirm the crystalline phase of the Mg_1−*x*_Cu*_x_*Al_2_O_4_ ceramics, X-ray diffraction (XRD, Miniflex600, Rigaku, Tokyo, Japan), using Cu K_α_ radiation (λ = 1.54 Å) at room temperature, was measured in the 2θ angle range between 10° and 120° with a step of 0.01°, and counting time for 5 s per step. Based on the XRD results, the crystal structure was analyzed using the Rietveld refinement method using FullProf software (FullProf Suite May2021 64b, The FullProf Team, Grenoble, France) [23]. The microstructures and morphologies of the samples were analyzed by a scanning electron microscope (SEM, JSM-6490; JEOL, Tokyo, Japan) at an accelerating voltage of 20 kV. The Al^3+^ ion distributions of Mg_1−*x*_Cu*_x_*Al_2_O_4_ were investigated through ^27^Al solid-state magic-angle spinning nuclear magnetic resonance (MAS-NMR) with a spinning frequency of 12 kHz (Avance II 600 MHz, Bruker, Fällanden, Switzerland).

Microwave dielectric properties (ε_r_, Qf, and τ_f_) of these samples were measured by the Hakki–Coleman dielectric resonator with a vector network analyzer (N5230A, Agilent Technologies, Santa Clara, CA, USA). The τ_f_ value was calculated based on the resonant frequencies at 25 and 85 °C:

(1) $$\tau_{f}=\frac{f_{85}-f_{25}}{60\times f_{25}}\times 10^{6}\left(\mathrm{ppm}/{}^{\circ}C\right)$$

where $f_{t}$ represents the resonant frequency at *t* °C.

## 3. Results and Discussion

**Figure 1:**
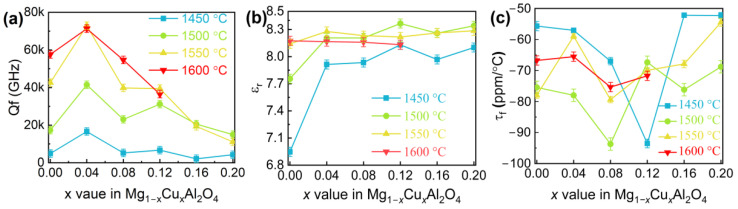
The microwave dielectric properties of Mg_1−*x*_Cu*_x_*Al_2_O_4_ (*x* = 0–0.2) ceramics sintered at 1450–1600 °C: (**a**) Qf value, (**b**) ε_r_ value, and (**c**) τ_f_ value.

Figure 1 shows the microwave dielectric properties of Mg_1−*x*_Cu*_x_*Al_2_O_4_ ceramics sintered at 1450–1600 °C. Good microwave dielectric properties were obtained at *x* = 0.04 with sintering at 1550 °C: ε_r_ = 8.28, Qf = 72,800 GHz, and τ_f_ = −59 ppm/°C. With the sintering temperature at 1550 °C, the τ_f_ value experienced a significant increase in the negative direction to about −59 ppm/°C at 0 ≤ *x* ≤ 0.04; then, the rapid fall was witnessed and a steady rise was observed at 0.08 ≤ *x* ≤ 0.20. Figure 1b shows the ε_r_ value, which increased first up to *x* = 0.12 and then presented a modest downward trend when the sintering temperatures were 1450 and 1500 °C, whereas it remained virtually unchanged at 1550 and 1600 °C. In addition, it is well known that Qf values are determined by both intrinsic and extrinsic factors. Intrinsic factor is mainly caused by lattice vibration, while extrinsic factor is dominated by grain boundary, secondary phase, and densification [24,25]. With the increase in the *x* value, the Qf values of the samples with different sintering temperatures increased initially and then showed a steady drop. The maximum Qf value was acquired at *x* = 0.04 with sintering at 1550 and 1600 °C. Compared with previous studies (see Table 1) [6,15,17,18,26,27,28,29,30,31,32,33], the sintering temperature and τ_f_ value of this work can be improved. However, Qf is lower than the best reported value [15], one of the reasons may be the different experimental conditions, such as preparation method, sintering temperature and ball milling time, etc. To understand the microstructure and microwave dielectric properties of Mg_1−*x*_Cu*_x_*Al_2_O_4_ ceramics, the phase composition, relative density, and cation distribution were investigated in this study.

**Figure 2:**
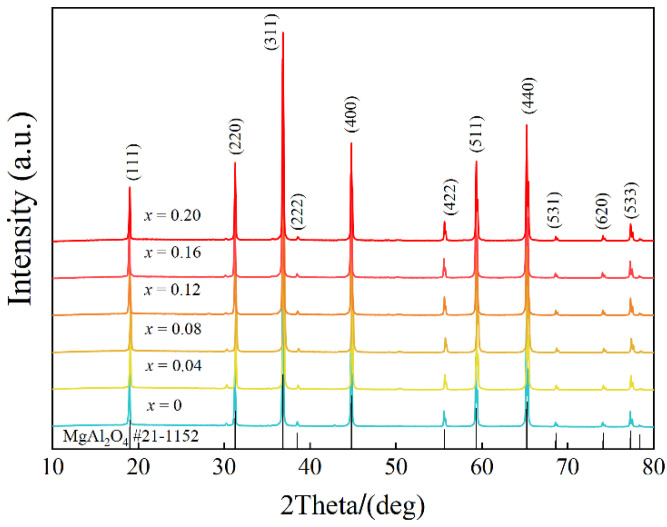
The XRD patterns of the Mg_1−*x*_Cu*_x_*Al_2_O_4_ ceramics.

XRD analysis is often carried out to identify phases. The XRD patterns of Mg_1−*x*_Cu*_x_*Al_2_O_4_ ceramics sintered at 1550 °C are illustrated in Figure 2. All the diffraction peaks can be assigned to those for the standard MgAl_2_O_4_ (PDF # 21-1125) pattern with a space group Fd-3m (227). Meanwhile, there is no apparent peak corresponding to any additional secondary phase containing Cu or structural phase transitions observed from Figure 2, indicating the successful formation of Mg_1−*x*_Cu*_x_*Al_2_O_4_ solid solutions [34].

**Figure 3:**
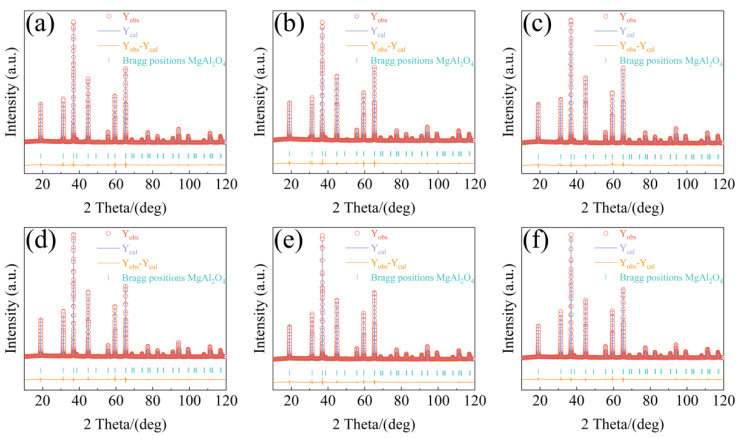
The XRD with Rietveld refinements: (**a**) *x* = 0, (**b**) *x* = 0.04, (**c**) *x* = 0.08, (**d**) *x* = 0.12, (**e**) *x* = 0.16, and (**f**) *x* = 0.20.

**Figure 4:**
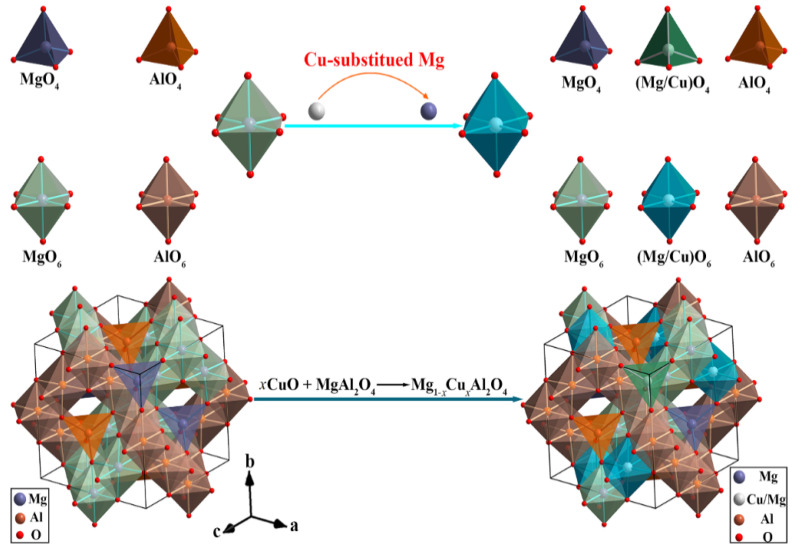
The schematic diagram of a crystal structure for MgAl_2_O_4_.

Figure 3 displays the Rietveld refinement of the XRD patterns for Mg_1−*x*_Cu*_x_*Al_2_O_4_ ceramics, and the refinement results are presented in Table 2. Both the Bragg positions models are well within the standard indexed peaks, indicating that the refinement result is acceptable. According to the refinement, the Cu^2+^ ions occupied the tetrahedral site, with the exception of a small amount of the octahedral site. Figure 4 shows the schematic diagram of a crystal structure from MgAl_2_O_4_ to Mg_1−*x*_Cu*_x_*Al_2_O_4_. The Mg^2+^ and Cu^2+^ ions significantly occupy the 8a Wyckoff position, and the Al^3+^ ions mainly occupy the 16d Wyckoff position. They form a [Mg(T)/Cu(T)O_4_] tetrahedron and an [Al(M)O_6_] octahedron, respectively. In general, Al^3+^ ions preferentially occupy tetrahedra, which can effectively improve the Qf value (~232,301 GHz) [19,26]. However, Al^3+^ ions mainly occupy the octahedron, which is consistent with low Qf values (~72,800 GHz) of the Mg_1−*x*_Cu*_x_*Al_2_O_4_ system. The cell parameters showed a nonlinear trend with an increase in Cu^2+^ ions content; that is, they first increased, then decreased, and finally increased. On the one hand, the radius of a Cu^2+^ ion (*r* = 0.73 Å) is slightly larger than that of a Mg^2+^ ion (*r* = 0.72 Å) [20], which may have led to the increase in cell parameters. On the other hand, the Cu^2+^ ions’ octahedral coordination has a significant Jahn–Teller effect. The Jahn–Teller distortion can enhance the polarizing effect of Cu^2+^ ions [22]. Therefore, the hybridization of Cu^2+^ ions is responsible for a decrease in average cell parameters [22]. Consequently, the two mechanisms were in competition with each other, resulting in a nonlinear variation trend of cell parameters. The cation distribution, which is a significant variation, had no obvious effect on the microwave dielectric properties. 

**Figure 5:**
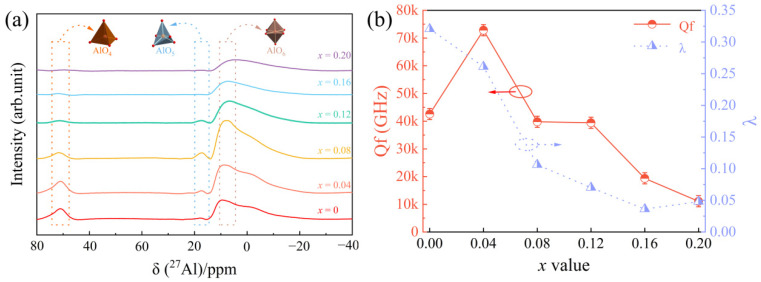
(**a**) ^27^Al NMR spectra of Mg_1−*x*_Cu*_x_*Al_2_O_4_ ceramics sintered at 1550 °C; (**b**) the effects of the degree of inversion on Qf value at 1550 °C.

For further analysis of the effects of the introduction of Cu^2+^ ions on microwave dielectric properties, the variations in the cation distribution in Al^3+^ were measured via the ^27^Al solid-state MAS-NMR measurement, which was used to evaluate the Al^3+^ sites in Mg_1−*x*_Cu*_x_*Al_2_O_4_ ceramics. Figure 5a shows the ^27^Al NMR spectra of Mg_1−*x*_Cu*_x_*Al_2_O_4_ ceramics sintered at 1550 °C. The spectra indicate three signals with chemical shifts at ca. 10, 17, and 71 ppm. They correspond to octahedrally coordinated aluminum (AlO_6_), pentahedrally coordinated aluminum (AlO_5_), and tetrahedrally coordinated aluminum (AlO_4_), respectively [35,36]. For the emergence of AlO_5_, a dynamic disorder occurred between the twisted tetrahedral structure and octahedral structure and froze some Al ions stuck in the pentahedral structure at high temperatures [37]. In Figure 5a, the peak intensities of AlO_4_ at 71 ppm gradually weakened, whereas the peaks of AlO_6_ at 10 ppm and AlO_5_ at 17 ppm first increased and then decreased with the increase in Cu^2+^ content. This result indicated that the redistribution of Al^3+^ ions in the lattice occurred by the substitution of Cu^2+^ for Mg^2+^. Moreover, the peaks transferred to low chemical shifts on account of the second-order quadrupolar-order shifts with the increase in Cu^2+^ content [31,38]. On the whole, the peak intensity of AlO_6_ is significantly larger than that of AlO_4_, which indicates that Al^3+^ ions mainly occupied octahedral sites. This was in accordance with the XRD results. The different peaks indicated the existence of an intermediate spinel structure in the system. The intermediate spinel structure can be described as [Mg_1−λ_^2+^Al_λ_^3+^]^IV^[Al_2−λ_^3+^Mg_λ_^2+^]^VI^O_4_, where λ is the degree of the inversion of spinel structure, corresponding to the fraction of Al^3+^ ions in the tetrahedral site. The value of λ ranges from 0 (normal spinel: (Mg^2+^)^IV^(Al_2_^3+^)^VI^O_4_) to 1 (inverse spinel: (Al^3+^)^IV^(Al^3+^Mg^2+^)^VI^O_4_) [13,14]. It is known that the microwave dielectric properties are related to λ [14,15,39]. The value of λ can be calculated with the following formula [40]:

(2) $$\lambda =\frac{2I\left({\mathrm{AlO}}_{4}\right)}{I\left({\mathrm{AlO}}_{4}\right)+I\left({\mathrm{AlO}}_{6}\right)}$$

where *I*(AlO_4_) and *I*(AlO_6_) are the intensities of tetrahedral and octahedral resonances, respectively. The λ values are displayed in Figure 5b. Considering the trend of λ in Figure 5b, the Al^3+^ cations preferentially occupied the octahedral sites. It has been reported that the preferential tetrahedron site occupation of Al^3+^ could enhance the Qf value of the system [19,26]. Consequently, when 0.04 < *x* < 0.16, the Qf values reduce with the decrease in the λ value in Mg_1−*x*_Cu*_x_*Al_2_O_4_ ceramics (see Figure 5b). In addition, the entry of Cu^2+^ ions into the MgAl_2_O_4_ lattice can lead to disordered charge distribution, which can cause a decrease in Qf value at high Cu^2+^ ions content [41].

**Figure 6:**
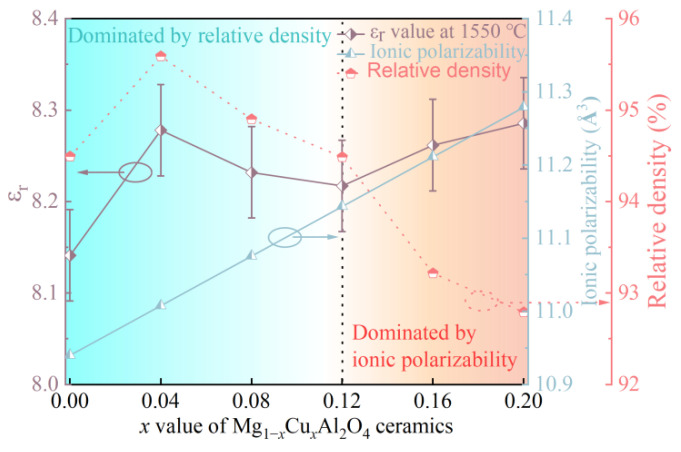
The variation between ε_r_ value at 1550 °C and theoretical ionic polarizabilities; the relative density.

The relative density can be calculated according to measured and theoretical density. The theoretical density can be derived from XRD refinements. The results are presented in Table 2. The relative density first increased and then gradually decreased, and the maximum value, which was obtained at *x* = 0.04, was 95.59%. The SEM shows that the densification was consistent with the relative density (see Figure S1). It also indicates that the moderate amount of CuO can promote the sintering of MgAl_2_O_4_ ceramics, which is beneficial for obtaining uniform and dense microstructures (see Figure S1). The relationship between the ε_r_ value and the relative density is presented in Figure 6, which shows that the ε_r_ value and relative density showed the same trend when *x* ≤ 0.12. With the increase in Cu^2+^ at *x* > 0.12, the pores in ceramics also played an important role for the ε_r_ value. To further investigate the effect of porosity on the ε_r_ value, the porosity-corrected dielectric constant (ε_rc_) can be calculated by a spherical-pore model [42]:

(3) $$\varepsilon_{r}=\varepsilon_{\mathrm{rc}}\left(1-\frac{3P\left(\varepsilon_{\mathrm{rc}}-1\right)}{2\varepsilon_{\mathrm{rc}}+1}\right)$$

where ε_r_ and *P* (1 − ρ_relative_) are the measured ε_r_ and the porosity, respectively. The calculated results are listed in Table 3. The ε_rc_ value was higher than the ε_r_ value, which indicated that the air trapped in the pores contributed to the decrease in the dielectric constant [43]. It is worth mentioning that, with the increase in Cu^2+^ at *x* > 0.12, the relative density maintained a declining trend, while the ε_r_ value had a slight growth. In response to this difference, apart from the relative density and pores, the variation in the ε_r_ value can be evaluated by the Clausius–Mosotti equation [44]:

(4) $$\varepsilon_{\mathrm{theo}}=\frac{3V_{m}+8\pi \alpha_{\mathrm{theo}}}{3V_{m}-4\pi \alpha_{\mathrm{theo}}}$$

where *V*_m_ and *α*_theo_ represent the molecular volume and theoretical ionic polarizabilities, respectively. *α*_theo_ can be calculated as follows [45,46]:

(5) $$\alpha_{\mathrm{theo}}=\left(1-x\right)\alpha \left(Mg^{2+}\right)+x\alpha \left(Cu^{2+}\right)+2\alpha \left(Al^{3+}\right)+4\alpha \left(O^{2-}\right)$$

where the *α*_i_ value corresponds to the individual ionic dielectric polarizabilities. The results are listed in Table 3. The theoretical ionic polarizabilities of Mg_1−*x*_Cu*_x_*Al_2_O_4_ ceramics, which increased linearly from 10.94 to 11.10, are shown in Figure 6. In general, the increase in polarizabilities led to the increase in the ε_r_ value, and the expected variation only occurred at *x* ≥ 0.12 [47]. This indicates that the ionic polarizabilities have a more significant impact on the ε_r_ value than that of the relative density at *x* > 0.12.

**Figure 7:**
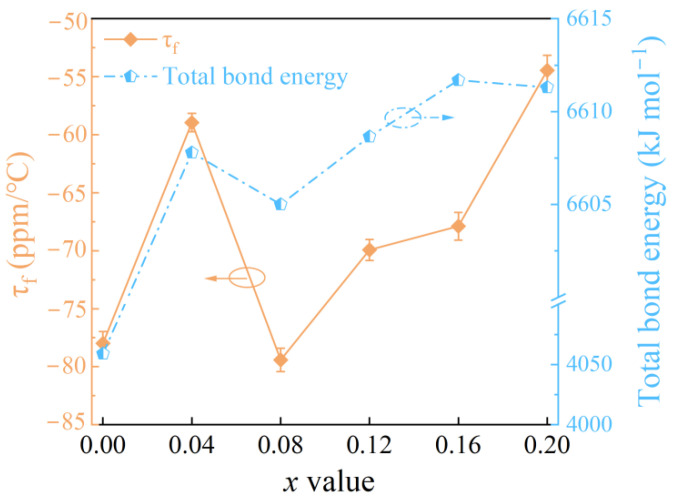
The τ_f_ value and the total bond energy of Mg_1−*x*_Cu*_x_*Al_2_O_4_ ceramics sintered at 1550 °C.

In order to clarify the correlation between the chemical bonds and the microwave dielectric properties of Mg_1−*x*_Cu*_x_*Al_2_O_4_ ceramics at 1550 °C, the complex chemical bond theory analysis was carried out, which was contributed by Phillips, Van Vecten, and Levine (P-V-L) [48,49,50,51,52]. The detailed process of the P-V-L theory analysis is presented in the Supplementary Materials. The bond length, lattice energy, and bond energy, which are calculated through P-V-L theory, are shown in Tables S1–S4, respectively. The τ_f_ value is the combined result of the bonding strength and the crystal structure. In general, the binding force between the ions in the unit cell was stronger, the restoring force that affected the tilt of the oxygen octahedron was higher, the unit cell was less affected at high temperatures, and the τ_f_ value was closer to zero [21,53]. Figure 7 shows the τ_f_ value and the total bond energy as a function of the *x* value. When *x* ≤ 0.04 and *x* ≥ 0.08, the τ_f_ value shifted to zero with the increase in total bond energy, indicating that the system tended to be stable. 

## 4. Conclusions

A single-phase Mg_1−*x*_Cu*_x_*Al_2_O_4_ ceramic with a spinel structure was formulated and analyzed. The Cu^2+^ ions occupied the tetrahedral site, whereas the Al^3+^ ions preferentially occupied octahedral site, resulting in a low the Qf value. In addition, the entry of Cu^2+^ ions into the MgAl_2_O_4_ lattice lead to disordered charge distribution, which can cause a decrease in Qf value at high Cu^2+^ ions content. The Cu substitution had the high bond energy, which contributed to the temperature stability of the samples at *x* ≤ 0.04 and *x* ≥ 0.08. Then, the τ_f_ value moved toward the positive direction. Good microwave dielectric properties were achieved at *x* = 0.04, sintered at 1550 °C: ε_r_ = 8.28, Qf = 72,800 GHz, and τ_f_ = −59 ppm/°C. Therefore, the Qf and τ_f_ values of the Mg_1__−*x*_Cu*_x_*Al_2_O_4_ solid solution were improved, maintaining a low ε_r_ value. This study suggests that Mg_1__−*x*_Cu*_x_*Al_2_O_4_ is a promising candidate ceramic, possessing a high Qf value and a low dielectric constant, for use in modern communication systems.

## Figures and Tables

**Table 1:** Microwave dielectric properties and preparation condition of MgAl_2_O_4_-based and ZnAl_2_O_4_ ceramics.

Sample	τ_f_ (ppm/°C)	Qf (GHz)	ε_r_	T_s_ (°C)	Milling Time (h)	Preparation Method	Ref.
Mg_0.96_Cu_0.04_Al_2_O_4_	−59	72,800	8.28	1550	4	solid state reaction	This work
MgAl_2_O_4_	N/A	82,000	7.9	1550–1700	24	solid state reaction	[26]
ZnAl_2_O_4_	N/A	106,000	8.6	1550–1700	24	solid state reaction	[26]
Mg(Al_0.4_Ga_0.6_)_2_O_4_	−16	107,000	8.87	1285–1535	6	solid state reaction	[27]
MgAl_1.94_(Mg_0.5_Ti_0.5_)_0.06_O_4_	−61.36	98,000	9.1	1425	6	solid state reaction	[28]
Mg_0.25_Zn_0.75_Al_2_O_4_	−60	222,600	8.40	1600	24	solid state reaction	[29]
0.75MgAl_2_O_4_-0.25TiO_2_	−12	105,400	11.04	1400–1460	24	solid state reaction	[6]
(Mg_0.75_Ni_0.25_)Al_2_O_4_	−53.5	130,000	8.21	1480–1600	24	solid state reaction	[17]
(Mg_0.95_Zn_0.05_)Al_2_O_4_	−64~−70	156,000	8.1	1480–1600	12	solid state reaction	[30]
Zn_0.4_Al_2.4_O_4_	−66	202,468	8.2	1500–1600	24	molten salt method	[31]
MgAl_2_O_4_	−62.4	201,690	7.8	1600	24	molten salt method	[15]
Mg_0.7_Al_2.2_O_4_	−60	201,111	7.7	1600	24	molten salt method	[32]
Mg_0.4_Al_2.4_O_4_	−60	232,301	7.5	1600	24	molten salt method	[32]
Mg_0.8_Co_0.2_Al_2_O_4_	−60	49,300	8.46	1475–1500	12	reaction-sintering process	[18]
Transparent MgAl_2_O_4_	N/A	52,640	8.20	1350	N/A	spark plasma sintering	[33]

Note: N/A is not applicable.

**Table 2:** The cell parameters, density, and reliable factors were obtained based on the Rietveld refinements of XRD.

	*x* = 0	*x* = 0.04	*x* = 0.08	*x* = 0.12	*x* = 0.16	*x* = 0.20
*a* = *b* = *c* (Å)	8.0849	8.0864	8.0869	8.0838	8.0817	8.0828
*V* (Å^3^)	528.467	528.775	528.857	528.259	527.841	528.060
*R*_p_ (%)	6.35	6.51	5.41	4.76	4.35	3.99
*R*_wp_ (%)	8.54	8.88	7.19	6.10	5.63	5.17
*R*_exp_ (%)	4.72	4.60	4.29	3.94	3.70	3.56
*χ* ^2^	3.27	3.73	2.81	2.40	2.32	2.10
ρ_m_ (g∙cm^−3^)	3.379	3.454	3.465	3.491	3.484	3.503
ρ_t_ (g∙cm^−3^)	3.577	3.614	3.652	3.696	3.738	3.776
ρ_r_ (%)	94.49	95.59	94.90	94.49	93.22	92.79

Note: The ρ_m_, ρ_t_, and ρ_r_ are the measured density, the measured density, and the relative density, respectively.

**Table 3:** Measured dielectric constant (ε_r_) and porosity-corrected dielectric constant (ε_rc_) of Mg_1−*x*_Cu*_x_*Al_2_O_4_ ceramics sintered at 1550 °C.

*x* Value	*x* = 0	*x* = 0.04	*x* = 0.08	*x* = 0.12	*x* = 0.16	*x* = 0.2
*P*	0.055	0.044	0.051	0.055	0.068	0.072
ε_r_	8.14	8.28	8.23	8.22	8.26	8.29
ε_rc_	8.75	8.76	8.80	8.83	9.04	9.12
ε_theo_	7.79	7.92	8.05	8.23	8.40	8.54

## Data Availability

Not applicable.

## Supplementary Materials

The following supporting information can be downloaded at https://www.mdpi.com/article/10.3390/nano12193332/s1, Figure S1: SEM micrograph for Mg_1−*x*_Cu*_x_*Al_2_O_4_ ceramics: (a) *x* = 0, 1550 °C; (b) *x* = 0.04, 1550 °C; (c) *x* = 0.12, 1550 °C; (d) *x* = 0.04, 1450 °C; (e) *x* = 0.04, 1500 °C; and (f) *x* = 0.04, 1600 °C. Table S1: Bond length d (Å) for Mg_1−*x*_Cu*_x_*Al_2_O_4_ ceramics sintered at 1550 °C;. Table S2: Parameters of lattice energy for Mg_1−*x*_Cu*_x_*Al_2_O_4_ ceramics sintered at 1550 °C;. Table S3: Lattice energy U_cal_ (kJ mol^−1^) for Mg_1−*x*_Cu*_x_*Al_2_O_4_ ceramics sintered at 1550 °C;. Table S4: Bond energy E (kJ mol^−1^) for Mg_1−*x*_Cu*_x_*Al_2_O_4_ ceramics sintered at 1550 °C;. References [21,51,54,55,56,57,58,59,60,61,62,63] were cited in the Supplementary Materials.

## References

[1] Zhang Y., Deng J.Y., Li M.J., Sun D., Guo L.X. (2019). A MIMO Dielectric Resonator Antenna with Improved Isolation for 5G Mm-Wave Applications. IEEE Antennas Wirel. Propag. Lett..

[2] Choudhary P., Kumar R., Gupta N. (2015). Dielectric Material Selection of Microstrip Patch Antenna for Wireless Communication Applications Using Ashby’s Approach. Int. J. Microw. Wirel. Technol..

[3] Zhang Y.P., Liu D. (2009). Antenna-on-Chip and Antenna-in-Package Solutions to Highly Integrated Millimeter-Wave Devices for Wireless Communications. IEEE Trans. Antennas Propag..

[4] Lai Y., Tang X., Huang X., Zhang H., Liang X., Li J., Su H. (2018). Phase Composition, Crystal Structure and Microwave Dielectric Properties of Mg_2−*x*_Cu*_x_*SiO_4_ Ceramics. J. Eur. Ceram. Soc..

[5] Hu X., Huang X.J., Chen Y.H., Li Y., Ling Z.Y. (2020). Phase Evolution and Microwave Dielectric Properties of SrTiO_3_ Added ZnAl_2_O_4_–Zn_2_SiO_4_–SiO_2_ Ceramics. Ceram. Int..

[6] Surendran K.P., Bijumon P.V., Mohanan P., Sebastian M.T. (2005). (1−*x*)MgAl_2_O_4_−*x*TiO_2_ Dielectrics for Microwave and Millimeter Wave Applications. Appl. Phys. A Mater. Sci. Process..

[7] Yu J., Shen C., Qiu T. (2015). Effect of Microwave Sintering on the Microstructure and Dielectric Properties of 0.92MgAl_2_O_4_–0.08(Ca_0.8_Sr_0.2_)TiO_3_ Ceramics. J. Mater. Sci. Mater. Electron..

[8] Nong L., Cao X., Li C., Liu L., Fang L., Khaliq J. (2021). Influence of Cation Order on Crystal Structure and Microwave Dielectric Properties in *x*Li_4/3_Ti_5/3_O_4_-(1−*x*)Mg_2_TiO_4_ (0.6 ≤ *x* ≤ 0.9) Spinel Solid Solutions. J. Eur. Ceram. Soc..

[9] Li H., Tang B., Li Y., Qing Z. (2015). Effects of Mg_2.05_SiO_4.05_ Addition on Phase Structure and Microwave Properties of MgTiO_3_-CaTiO_3_ Ceramic System. Mater. Lett..

[10] Jia X., Xu Y., Zhao P., Li J., Li W. (2021). Structural Dependence of Microwave Dielectric Properties in Ilmenite-Type Mg(Ti_1−*x*_Nb*_x_*)O_3_ Solid Solutions by Rietveld Refinement and Raman Spectra. Ceram. Int..

[11] Liao Q., Li L., Ding X. (2012). Phase Constitution, Structure Analysis and Microwave Dielectric Properties of Zn 0.5Ti_1−*x*_Zr*_x_*NbO_4_ Ceramics. Solid State Sci..

[12] Bruschini E., Speziale S., Bosi F., Andreozzi G.B. (2018). Fe–Mg Substitution in Aluminate Spinels: Effects on Elastic Properties Investigated by Brillouin Scattering. Phys. Chem. Miner..

[13] Millard R.L., Peterson R.C., Hunter B.K. (1992). Temperature Dependence of Cation Disorder in MgAl_2_O_4_ Spinel Using ^27^Al and ^17^O Magic-Angle Spinning NMR. Am. Mineral..

[14] Kan A., Okazaki H., Takahashi S., Ogawa H. (2018). Microwave Dielectric Properties and Cation Distribution of Spinel-Structured Mg_0.4_Al_2.4−*x*_Ga*_x_*O_4_ Ceramics with Cation Defect. Jpn. J. Appl. Phys..

[15] Takahashi S., Kan A., Ogawa H. (2017). Microwave Dielectric Properties and Crystal Structures of Spinel-Structured MgAl_2_O_4_ Ceramics Synthesized by a Molten-Salt Method. J. Eur. Ceram. Soc..

[16] Qin T., Zhong C., Shang Y., Cao L., Wang M., Tang B., Zhang S. (2021). Effects of LiF on Crystal Structure, Cation Distributions and Microwave Dielectric Properties of MgAl_2_O_4_. J. Alloys Compd..

[17] Huang C.L., Tai C.Y., Huang C.Y., Chien Y.H. (2010). Low-Loss Microwave Dielectrics in the Spinel-Structured (Mg_1−*x*_Ni*_x_*)Al_2_O_4_ Solid Solutions. J. Am. Ceram. Soc..

[18] Tsai W.C., Liou Y.H., Liou Y.C. (2012). Microwave Dielectric Properties of MgAl_2_O_4_-CoAl_2_O_4_ Spinel Compounds Prepared by Reaction-Sintering Process. Mater. Sci. Eng. B Solid-State Mater. Adv. Technol..

[19] Takahashi S., Ogawa H., Kan A. (2018). Electronic States and Cation Distributions of MgAl_2_O_4_ and Mg_0.4_Al_2.4_O_4_ Microwave Dielectric Ceramics. J. Eur. Ceram. Soc..

[20] Shannon R.D. (1976). Revised Effective Ionic Radii and Systematic Studies of Interatomie Distances in Halides and Chaleogenides. Acta Crystallogr. Sect. A.

[21] Lai Y., Zeng Y., Han J., Liang X., Zhong X., Liu M., Duo B., Su H. (2021). Structure Dependence of Microwave Dielectric Properties in Zn_2−*x*_SiO_4−*x*_-xCuO Ceramics. J. Eur. Ceram. Soc..

[22] Le Nestour A., Gaudon M., Villeneuve G., Andriessen R., Demourgues A. (2007). Steric and Electronic Effects Relating to the Cu^2+^ Jahn-Teller Distortion in Zn_1−*x*_Cu*_x_*Al_2_O_4_ Spinels. Inorg. Chem..

[23] Rodríguez-Carvajal J. (1993). Recent Advances in Magnetic Structure Determination by Neutron Powder Diffraction. Phys. B Phys. Condens. Matter.

[24] Kim J.M., Jo H.W., Kim E.S. (2019). Effect of Electronegativity on Microwave Dielectric Properties of MgTi_1−*x*_(A_1/3_Sb_2/3_)*_x_*O_3_ (A = Mg^2+^, Zn^2+^) Ceramics. Int. J. Appl. Ceram. Technol..

[25] Lai Y., Su H., Wang G., Tang X. (2019). Low-Temperature Sintering of Microwave Ceramics with High Qf Values through LiF Addition. J. Am. Ceram. Soc..

[26] Zheng C.W., Fan X.C., Chen X.M. (2008). Analysis of Infrared Reflection Spectra of (Mg_1−*x*_Zn*_x_*)Al_2_O_4_ Microwave Dielectric Ceramics. J. Am. Ceram. Soc..

[27] Wu S., Xue J., Fan Y. (2014). Spinel Mg(Al, Ga)_2_O_4_ Solid Solution as High-Performance Microwave Dielectric Ceramics. J. Am. Ceram. Soc..

[28] Qin T., Zhong C., Qin Y., Tang B., Zhang S. (2020). The Structure Evolution and Microwave Dielectric Properties of MgAl_2−*x*_(Mg_0.5_Ti_0.5_)*_x_*O_4_ Solid Solutions. Ceram. Int..

[29] Takahashi S., Kan A., Ogawa H. (2017). Effects of Cation Distribution on Microwave Dielectric Properties of Mg_1−*x*_Zn*_x_*Al_2_O_4_ Ceramics. Mater. Chem. Phys..

[30] Huang C.L., Chien Y.H., Tai C.Y., Huang C.Y. (2011). High-Q Microwave Dielectrics in the (Mg_1−*x*_Zn*_x_*)Al_2_O_4_ (x = 0–0.1) System. J. Alloys Compd..

[31] Takahashi S., Kan A., Ogawa H. (2017). Microwave Dielectric Properties and Cation Distributions of Zn_1−3*x*_Al_2+2*x*_O_4_ Ceramics with Defect Structures. J. Eur. Ceram. Soc..

[32] Takahashi S., Kan A., Ogawa H. (2017). Microwave Dielectric Properties and Crystal Structures of Mg_0.7_Al_2.2_O_4_ and Mg_0.4_Al_2.4_O_4_ Ceramics with Defect Structures. J. Am. Ceram. Soc..

[33] Fu P., Xu Y., Shi H., Zhang B., Ruan X., Lu W. (2014). The Effect of Annealing Process on the Optical and Microwave Dielectric Properties of Transparent MgAl_2_O_4_ Ceramics by Spark Plasma Sintering. Opt. Mater. (Amst.).

[34] Fregola R.A., Bosi F., Skogby H., Hålenius U. (2012). Cation Ordering over Short-Range and Long-Range Scales in the MgAl_2_O_4_-CuAl_2_O_4_ Series. Am. Mineral..

[35] Khanna A., Saini A., Chen B., González F., Pesquera C. (2013). Structural Study of Bismuth Borosilicate, Aluminoborate and Aluminoborosilicate Glasses by ^11^B and ^27^Al MAS NMR Spectroscopy and Thermal Analysis. J. Non. Cryst. Solids.

[36] MacKenzie K.J.D., Smith M.E. (2002). Chapter 5: ^27^Al NMR. Pergamon Materials Series.

[37] Harindranath K., Anusree Viswanath K., Vinod Chandran C., Bräuniger T., Madhu P.K., Ajithkumar T.G., Joy P.A. (2010). Evidence for the Co-Existence of Distorted Tetrahedral and Trigonal Bipyramidal Aluminium Sites in SrAl1_2_O_19_ from ^27^Al NMR Studies. Solid State Commun..

[38] Pellerin N., Dodane-Thiriet C., Montouillout V., Beauvy M., Massiot D. (2007). Cation Sublattice Disorder Induced by Swift Heavy Ions in MgAl_2_O_4_ and ZnAl_2_O_4_ Spinels: ^27^Al Solid-State NMR Study. J. Phys. Chem. B.

[39] Takahashi S., Imai Y., Kan A., Hotta Y., Ogawa H. (2015). Microwave Dielectric Properties of Composites Consisting of MgAl_2_O_4_ Filler Synthesized by Molten-Salt Method and Isotactic Polypropylene Polymer Matrix. Jpn. J. Appl. Phys..

[40] Blaakmeer E.S., Rosciano F., Van Eck E.R.H. (2015). Lithium Doping of MgAl_2_O_4_ and ZnAl_2_O_4_ Investigated by High-Resolution Solid State NMR. J. Phys. Chem. C.

[41] Tamura H. (2006). Microwave Dielectric Losses Caused by Lattice Defects. J. Eur. Ceram. Soc..

[42] Santhosh Kumar T., Pamu D. (2015). Effect of V_2_O_5_ on Microwave Dielectric Properties of Non-Stoichiometric MgTiO_3_ Ceramics. Mater. Sci. Eng. B Solid-State Mater. Adv. Technol..

[43] Sayyadi-Shahraki A., Taheri-Nassaj E., Hassanzadeh-Tabrizi S.A., Barzegar-Bafrooei H. (2014). A New Temperature Stable Microwave Dielectric Ceramic with Low-Sintering Temperature in Li_2_TiO_3_-Li_2_Zn_3_Ti _4_O_12_ System. J. Alloys Compd..

[44] Wang F., Lai Y., Zeng Y., Yang F., Li B., Yang X., Su H., Han J., Zhong X. (2021). Enhanced Microwave Dielectric Properties in Mg_2_Al_4_Si_5_O_18_ Through Cu^2+^ Substitution. Eur. J. Inorg. Chem..

[45] Shannon R.D. (1993). Dielectric Polarizabilities of Ions in Oxides and Fluorides. J. Appl. Phys..

[46] Zhang X., Tang B., Fang Z., Yang H., Xiong Z., Xue L., Zhang S. (2018). Structural Evolution and Microwave Dielectric Properties of a Novel Li_3_Mg_2−*x*/3_Nb_1−2*x*/3_Ti*_x_*O_6_ System with a Rock Salt Structure. Inorg. Chem. Front..

[47] Xiao E.C., Shi F., Fu G., Ren Q., Dou G., Lei W., Qi Z.M. (2020). Effects of BaCu(B_2_O_5_) Additives on the Crystal Structures and Dielectric Properties of CaMgGeO_4_ ceramics for LTCC Applications. CrystEngComm.

[48] Levine B.F. (1973). D-Electron Effects on Bond Susceptibilities and Ionicities. Phys. Rev. B.

[49] Phillips J.C., Van Vechten J.A. (1969). Dielectric Classification of Crystal Structures, Ionization Potentials, and Band Structures. Phys. Rev. Lett..

[50] Levine B.F. (1973). Bond Susceptibilities and Ionicities in Complex Crystal Structures. J. Chem. Phys..

[51] Wu Z., Meng Q. (1998). Semiempirical Study on the Valences of Cu and Bond Covalency in Y_1−*x*_Ca*_x_*Ba_2_Cu_3_O_6+y_. Phys. Rev. B-Condens. Matter Mater. Phys..

[52] Yang H., Zhang S., Yang H., Li E. (2020). Usage of P-V-L Bond Theory in Studying the Structural/Property Regulation of Microwave Dielectric Ceramics: A Review. Inorg. Chem. Front..

[53] Xiao M., Lou J., Zhou Z., Gu Q., Wei Y., Zhang P. (2017). Crystal Structure and Microwave Dielectric Properties of Ta^5+^ Substituted MgZrNb_2_O_8_ Ceramics. Ceram. Int..

[54] Xia W., Li L., Ning P., Liao Q. (2012). Relationship Between Bond Ionicity, Lattice Energy, and Microwave Dielectric Properties of Zn(Ta_1−*x*_Nb*_x_*)_2_O_6_ Ceramics. J. Am. Ceram. Soc..

[55] Van Vechten J.A. (1969). Quantum Dielectric Theory of Electronegativity in Covalent Systems. I. Electronic Dielectric Constant. Phys. Rev. (Ser. I).

[56] Zhang P., Zhao Y., Wang X. (2015). The Relationship Between Bond Ionicity, Lattice Energy, Coefficient of Thermal Expansion and Microwave Dielectric Properties of Nd(Nb_1−*x*_Sb*_x_*)O_4_ Ceramics. Dalton Trans..

[57] Zhang P., Zhao Y., Li L. (2015). The Correlations Among Bond Ionicity, Lattice Energy and Microwave Dielectric Properties of (Nd_1−*x*_La*_x_*)NbO_4_ Ceramics. Phys. Chem. Chem. Phys..

[58] Li C., Ding S., Zhang Y., Zhu H., Song T. (2020). Effects of Ni^2+^ Substitution on the Crystal Structure, Bond Valence, and Microwave Dielectric Properties of BaAl_2–2*x*_Ni_2*x*_Si_2_O_8–*x*_ Ceramics. J. Eur. Ceram. Soc..

[59] Sanderson R.T. (1983). Electronegativity and Bond Energy. J. Am. Chem. Soc..

[60] Xiao M., Wei Y., Zhang P. (2019). The Correlations between Complex Chemical Bond Theory and Microwave Dielectric Properties of Ca_2_MgSi_2_O_7_ Ceramics. J. Electron. Mater..

[61] Xiao M., Sun H., Zhou Z., Zhang P. (2018). Bond Ionicity, Lattice Energy, Bond Energy, and Microwave Dielectric Properties of Ca_1−*x*_Sr*_x_*WO_4_ Ceramics. Ceram. Int..

[62] Sanderson R. (1968). Multiple and Single Bond Energies in Inorganic Molecules. J. Inorg. Nucl. Chem..

[63] Luo Y.-R. (2007). Comprehensive Handbook of Chemical Bond Energies.

