# Supplementary material for: Cu2+-Ion-Substitution-Driven Microstructure and Microwave Dielectric Properties of Mg1−xCuxAl2O4 Ceramics
Source: Nanomaterials (Basel). 2022 Sep 24;12(19):3332. doi: 10.3390/nano12193332 (PMC9565854; doi:10.3390/nano12193332)
Supplement: Supplementary file 1 [file nanomaterials-12-03332-s001.zip › nanomaterials-1895270-supplementary.pdf]

nanomaterials

MDPI

Supplementary Materials

Cu^{2+}-Ion-Substitution-Driven Microstructure and Microwave Dielectric Properties of $Mg_{1-x}Cu_xAl_2O_4$ Ceramics

Yuanming Lai [1], Ming Yin [1,*], Baoyang Li [1], Xizhi Yang [1], Weiping Gong [2], Fan Yang [1], Qin Zhang [3], Fanshuo Wang [1], Chongsheng Wu [1] and Haijian Li [4]

[1] School of Mechanical and Electrical Engineering, Chengdu University of Technology, Chengdu 610059, China
[2] Guangdong Provincial Key Laboratory of Electronic Functional Materials and Devices, Huizhou University, Huizhou 516001, China
[3] State Key Laboratory of Electronic Thin Films and Integrated Devices, University of Electronic Science and Technology of China, Chengdu 610054, China
[4] Science and Technology on Combustion and Explosion Laboratory, Xi'an Modern Chemistry Research Institute, Xi'an 710065, China
* Correspondence: yinming2014@cdut.edu.cn

SEM analyses are carried out to confirm the microstructure of $Mg_{1-x}Cu_xAl_2O_4$ ceramics. Figure S1 presents the SEM for surfaces of the samples. Compared with the different CuO contents, in the SEM patterns at 1550 °C, there are many inhomogeneous and irregular grains at $x = 0$ (Figure S1a), and many pores with small punctate substance are observed at $x = 0.12$ (Figure S1c). When it comes to the influence of sintering temperature, there are many pores in the samples at 1450 °C and 1500 °C (Figure S1d,e), and incomplete grain growth is also observed. With a further increase in sintering temperature to 1550 °C and 1600 °C (Figure S1b,f), the SEM patterns comparatively present a more uniform and well-densified microstructure with little pores. The relative density reflects the densification of the microstructure. The optimal relative density corresponds to a uniform and compact microstructure formed at $x = 0.04$ and sintered at 1550 °C, as can be further demonstrated by the SEM images (Figure S1b). The maximum Qf value is 72 800 GHz at $x = 0.04$ sintered at 1550 °C, which corresponds to the high relative density and the uniform and compact microstructure. The Qf value of the $x = 0.04$ sample presents dramatic growth, with the increase in sintering temperature. This indicates that substitution with an appropriate amount of Cu^{2+} and increasing sintering temperature can improve the grain growth and densification in a $Mg_{1-x}Cu_xAl_2O_4$ solid solution, and the microstructure plays a significant role at the Qf value in $Mg_{1-x}Cu_xAl_2O_4$ ceramics

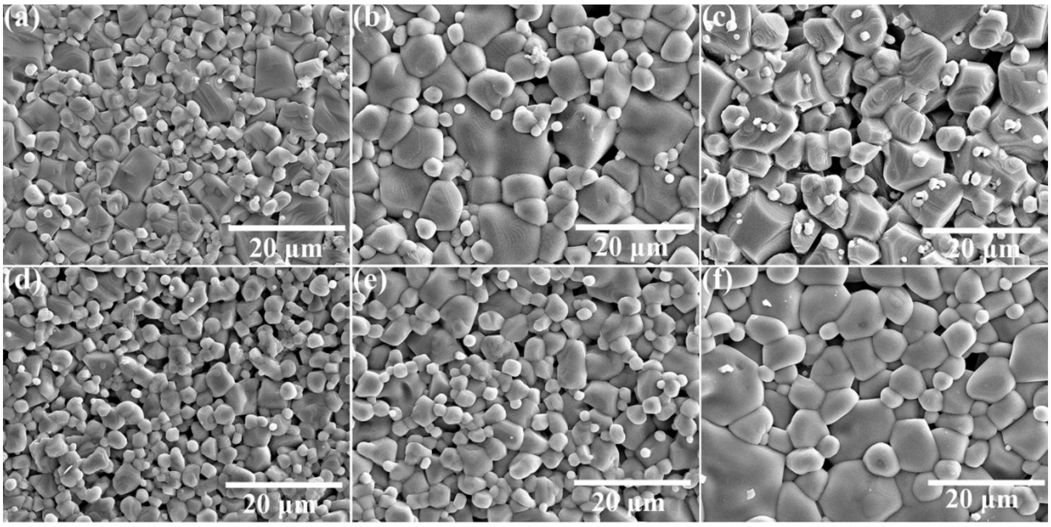

Figure S1. SEM micrograph for $Mg_{1-x}Cu_xAl_2O_4$ ceramics: (**a**) x = 0, 1550 °C; (**b**) x = 0.04, 1550 °C; (**c**) x = 0.12, 1550 °C; (**d**) x = 0.04, 1450 °C; (**e**) x = 0.04, 1500 °C; and (**f**) x = 0.04, 1600 °C.

The complex crystal structure can be transformed into binary crystal structure. The chemical formula for any complex crystal is as follows:

$$A^1{}_{a1}A^2{}_{a2}A^3{}_{a3}\ldots\ldots A^i{}_{ai}B^1{}_{b1}B^2{}_{b2}B^3{}_{b3}\ldots\ldots B^j{}_{bj} \quad (1)$$

where A is the cation and B is the anion. A^i and B^j represent different elements or occupy different units of the same element. a_i and b_j are the number of elements. Thus, based on the complex chemical bond theory analysis and the crystal structure of $Mg_{1-x}Cu_xAl_2O_4$, it can be decomposed into the sum of binary crystal structure as the following structures:

$$MgAl_2O_4 = Mg_{17/25}(T)O + Mg_{8/25}(M)O + Al_{8/25}(T)O + Al_{42/25}(M)O \quad (2)$$

Two kinds of chemical bonds—Mg-O and Al-O—exist in $MgAl_2O_4$ ceramics. Mg and Al ions have different coordination numbers. Compared with the crystal formula, each sub-formula in Equation (2) is more comprehensive in the charge balance. The effective valence electron numbers of the cations in the above bond are Z_{Mg} = 2, and Z_{Al} = 3. However, the effective valence electron numbers of the oxygen anions are different, which are determined by the different bond types in the sub-formula. In this work, the effective valence of the O^{2-} anions are Z_O = -1 in the Mg-O bond and Z_O = -3 in the Al-O bond. The difference in O^{2-} anions is due to the charge balance for each sub-formula. The characteristics of chemical bonds can be evaluated by covalency (f_c) and ionicity (f_i).

$$f_c^\mu = \left(\frac{E_h^\mu}{E_g^\mu}\right)^2 \quad (3)$$

$$f_i^\mu = \left(\frac{C^\mu}{E_g^\mu}\right)^2 \quad (4)$$

where the E_h^μ, E_g^μ, and C^μ are the homopolar part, average energy gap, and heteropolar component, respectively [1], and E_h^μ can be separated into E_g^μ and C^μ. On the basis of the previous reports [2–6], the lattice energy (U_{cal}) could be calculated using the chemical bond theory analysis according to the following Equations (5)-(8).

$$U_{cal} = \sum_\mu U_b^\mu \quad (5)$$

$$U_b^\mu = U_{bc}^\mu + U_{bi}^\mu \quad (6)$$

$$U_{bc}^\mu = 2100m\frac{\left(Z_+^\mu\right)^{1.64}}{\left(d^\mu\right)^{0.75}}f_c^\mu \quad (7)$$

$$U_{bi}^\mu = 1270\frac{(m+n)Z_+^\mu Z_-^\mu}{d^\mu}\left(1-\frac{0.4}{d^\mu}\right)f_i^\mu \quad (8)$$

where U_{bc}^μ is the covalent part of the μ bond and U_{bi}^μ is the ionic part of the μ bond. Z_+^μ and Z_-^μ are the valence states of cations and anions of the μ bond in the binary structure. d^μ is the bond length of μ bond (see Table S1). The parameters involved in the calculation are mentioned in Table S2. In addition, the lattice energy of $Mg_{1-x}Cu_xAl_2O_4$ ceramics sintered at 1550 °C is calculated (see Table S3). Based on previous reports [3,7], the total lat-

tice energy had a significant effect on the vibrational energy of ions and structural stability, which was an intrinsic factor in affecting Qf value.

It is similar to the relationship between Qf and lattice energy; the τ_f value is related to the bond energy, which indicates the stability of the crystal lattice [4]. The high bond energy corresponds to the stable vibration of the ions in the crystal lattice. Therefore, the relationships between τ_f value and bond energy are investigated in this work. Based on previous reports [8–11], the bond energy E can be calculated using the covalent radius, electronegativity, and bond length:

$$E = \sum_{\mu} E_b^{\mu} \tag{9}$$

$$E_b^{\mu} = t_c E_c^{\mu} + t_i E_i^{\mu} \tag{10}$$

$$E_i^{\mu} = \frac{33200}{d^{\mu}} \tag{11}$$

$$E_c^{\mu} = \frac{(r_{cA} + r_{cB})}{d^{\mu}} (E_{A-A} \times E_{B-B})^{1/2} \tag{12}$$

$$t_i + t_c = 1 \tag{13}$$

$$t_i = \left| \frac{S_A - S_B}{6} \right| \tag{14}$$

where E_b^{μ} is the individual bond energy of μ bond; E_i^{μ} is the unit charge product divided by the bond length d^{μ}; r_{cA} and r_{cB} are the covalent radii for cations and anions, respectively; $E_{A\text{-}A}$ and $E_{B\text{-}B}$ are the homonuclear bond energies; and S_A and S_B are the electronegativities of A and B ions. Here, $E_{\mathrm{O\text{-}O}}$ = 498.36 kJ mol^{-1}, $E_{\mathrm{Mg\text{-}Mg}}$ = 8.546 kJ mol^{-1}, $E_{\mathrm{Cu\text{-}Cu}}$ = 195.7 kJ mol^{-1}, $E_{\mathrm{Al\text{-}Al}}$ = 264.3 kJ mol^{-1} [12]. $r_{c\mathrm{O}}$ =63 pm, $r_{c\mathrm{Mg}}$ =139 pm, $r_{c\mathrm{Cu}}$ = 112 pm, $r_{c\mathrm{Al}}$ = 126 pm. S_{O} = 3.44, S_{Mg} = 1.31, S_{Cu} = 1.90, and S_{Al} = 1.61. The bond energies of the samples are shown in Table S4.

Table S1. Bond length d (Å) for $Mg_{1-x}Cu_xAl_2O_4$ ceramics sintered at 1550 °C.

Bond type	x = 0	x = 0.04	x = 0.08	x = 0.12	x = 0.16	x = 0.2
Mg(T)-O	1.908	1.922	1.927	1.924	1.920	1.917
Mg(M)-O	1.935	1.928	1.925	1.926	1.928	1.929
Al(T)-O	1.908	1.922	1.927	1.924	1.920	1.917
Al(M)-O	1.935	1.928	1.925	1.926	1.928	1.929
Cu(T)-O	/	1.922	1.927	1.924	1.920	1.917
Cu(M)-O	/	1.928	1.925	1.926	1.928	1.929

Note: T and M stand for tetrahedrally and octahedrally coordinated site, respectively.

Table S2. Parameters of lattice energy for $Mg_{1-x}Cu_xAl_2O_4$ ceramics sintered at 1550 °C.

x	Bond type	N_e^μ (10^{30}/m^3)	k_F^μ	E_h^μ (eV)	C^μ (eV)	f_c^μ (%)
x=0	Mg(T)-O	0.5419	2.5222	2.8295	−8.1995	10.64
	Mg(M)-O	0.2782	2.0195	2.7814	−12.6411	4.62
	Al(T)-O	0.5241	2.4942	2.8295	−12.4610	4.90
	Al(M)-O	1.4638	3.5126	2.7814	−22.1502	1.55
x=0.04	Mg(T)-O	0.5581	2.5471	2.8035	−7.9047	11.17
	Mg(M)-O	0.2486	1.9452	2.7936	−12.8716	4.50
	Al(T)-O	0.4628	2.3929	2.8035	−12.7566	4.61
	Al(M)-O	1.5173	3.5549	2.7936	−23.0069	1.45
	Cu(T)-O	0.5581	2.5471	2.8035	−7.9047	11.17
	Cu(M)-O	0.2486	1.9452	2.7936	−12.8716	4.50
x=0.08	Mg(T)-O	0.6296	2.6515	2.7936	−7.4583	12.30
	Mg(M)-O	0.1716	1.7192	2.7978	−13.2479	4.27
	Al(T)-O	0.3422	2.1640	2.7936	−14.1535	3.75
	Al(M)-O	1.6365	3.6457	2.7978	−24.5745	1.28
	Cu(T)-O	0.6296	2.6515	2.7936	−7.4583	12.30
	Cu(M)-O	0.1716	1.7192	2.7978	−13.2479	4.27
x=0.12	Mg(T)-O	0.6551	2.6869	2.8005	−7.3896	12.56
	Mg(M)-O	0.1510	1.6473	2.7963	−13.3511	4.20
	Al(T)-O	0.3137	2.1021	2.8005	−14.7019	3.50
	Al(M)-O	1.6680	3.6689	2.7963	−24.9166	1.24
	Cu(T)-O	0.6551	2.6869	2.8005	−7.3896	12.56
	Cu(M)-O	0.1510	1.6473	2.7963	−13.3511	4.20
x=0.16	Mg(T)-O	0.6757	2.7147	2.8081	−7.3520	12.73
	Mg(M)-O	0.1353	1.5883	2.7939	−13.4369	4.14
	Al(T)-O	0.2926	2.0538	2.8081	−15.1780	3.31
	Al(M)-O	1.6893	3.6845	2.7939	−25.1494	1.22
	Cu(T)-O	0.6757	2.7147	2.8081	−7.3520	12.73
	Cu(M)-O	0.1353	1.5883	2.7939	−13.4369	4.14
x=0.2	Mg(T)-O	0.6740	2.7125	2.8128	−7.3933	12.64
	Mg(M)-O	0.1403	1.6074	2.7905	−13.3677	4.18
	Al(T)-O	0.3020	2.0756	2.8128	−15.0703	3.37
	Al(M)-O	1.6787	3.6768	2.7905	−24.9788	1.23
	Cu(T)-O	0.6740	2.7125	2.8128	−7.3933	12.64
	Cu(M)-O	0.1403	1.6074	2.7905	−13.3677	4.18

Table S3. Lattice energy U_{cal} (kJ mol^{-1}) for $Mg_{1-x}Cu_xAl_2O_4$ ceramics sintered at 1550 °C.

**Bond Type**	***x* = 0**	***x* = 0.04**	***x* = 0.08**	***x* = 0.12**	***x* = 0.16**	***x* = 0.2**
Mg(T)-O	−7425.87*	−7918.75	−9725.02	−9907.51	−9909.98	−9314.87
Mg(M)-O	−4681.61	−3494.02	−1244.89	−729.50	−386.33	−463.91
Al(T)-O	−7115.61	−5512.74	−2080.58	−1284.15	−717.05	−904.86
Al(M)-O	−123429.67	−131284.08	−151033.20	−156421.36	−160472.27	−158959.99
Cu(T)-O		−329.95	−845.65	−1351.02	−1887.62	−2328.72
Cu(M)-O		−213.39	−108.25	−99.48	−73.59	−115.98
Total	−142652.76	−148752.93	−165037.59	−169793.01	−173446.83	−172088.33

* The negative sign of lattice energy represents the exothermic process.

Table S4. Bond energy E (kJ mol^{-1}) for $Mg_{1-x}Cu_xAl_2O_4$ ceramics sintered at 1550 °C.

Bond Type	*x* = 0	*x* = 0.04	*x* = 0.08	*x* = 0.12	*x* = 0.16	*x* = 0.2
Mg(T)-O	1211.73	1202.76	1199.31	1201.72	1204.34	1205.95
Mg(M)-O	1792.62	1798.97	1801.15	1800.40	1799.16	1797.38
Al(T)-O	425.35	422.20	420.99	421.84	422.76	423.32
Al(M)-O	629.26	631.49	632.25	631.99	631.55	630.93
Cu(T)-O	0.00	1022.70	1019.77	1021.82	1024.05	1025.42
Cu(M)-O		1529.66	1531.52	1530.88	1529.82	1528.31
Total	4058.97	6607.79	6605.00	6608.65	6611.69	6611.30

References

1. Xia, W.; Li, L.; Ning, P.; Liao, Q. Relationship Between Bond Ionicity, Lattice Energy, and Microwave Dielectric Properties of $Zn(Ta_{1-x}Nb_x)_2O_6$ Ceramics. *J. Am. Ceram. Soc.* **2012**, *95*, 2587–2592, https://doi.org/10.1111/j.1551-2916.2012.05231.x.
2. Van Vechten, J.A. Quantum Dielectric Theory of Electronegativity in Covalent Systems. I. Electronic Dielectric Constant. *Phys. Rev. (Series I)* **1969**, *182*, 891–905, https://doi.org/10.1103/physrev.182.891.
3. Zhang, P.; Zhao, Y.; Wang, X. The Relationship Between Bond Ionicity, Lattice Energy, Coefficient of Thermal Expansion and Microwave Dielectric Properties of $Nd(Nb_{1-x}Sb_x)O_4$ Ceramics. *Dalton Trans.* **2015**, *44*, 10932–10938, https://doi.org/10.1039/c5dt01343g.
4. Zhang, P.; Zhao, Y.; Li, L. The Correlations Among Bond Ionicity, Lattice Energy and Microwave Dielectric Properties of $(Nd_{1-x}La_x)NbO_4$ Ceramics. *Phys. Chem. Chem. Phys.* **2015**, *17*, 16692–16698, https://doi.org/10.1039/c5cp02204e.
5. Lai, Y.; Zeng, Y.; Han, J.; Liang, X.; Zhong, X.; Liu, M.; Duo, B.; Su, H. Structure dependence of microwave dielectric properties in $Zn_{2-x}SiO_{4-x}$-xCuO ceramics. *J. Eur. Ceram. Soc.* **2020**, *41*, 2602–2609, https://doi.org/10.1016/j.jeurceramsoc.2020.12.013.
6. Wu, Z.J.; Meng, Q.B.; Zhang, S.Y. Semiempirical Study on the Valences of Cu and Bond Covalency in $Y_{1-x}Ca_xBa_2Cu_3O_{6+y}$. *Phys. Rev. B* **1998**, *58*, 958–962, https://doi.org/10.1103/physrevb.58.958.
7. Li, C.; Ding, S.; Zhang, Y.; Zhu, H.; Song, T. Effects of Ni^{2+} Substitution on the Crystal Structure, Bond Valence, and Microwave Dielectric Properties of $BaAl_{2-2x}Ni_{2x}Si_2O_{8-x}$ Ceramics. *J. Eur. Ceram. Soc.* **2020**, *41*, 2610–2616, https://doi.org/10.1016/j.jeurceramsoc.2020.12.011.
8. Sanderson, R.T. Electronegativity and Bond Energy. *J. Am. Chem. Soc.* **1983**, *105*, 2259–2261, https://doi.org/10.1021/ja00346a026.
9. Xiao, M.; Wei, Y.; Zhang, P. The Correlations between Complex Chemical Bond Theory and Microwave Dielectric Properties of $Ca_2MgSi_2O_7$ Ceramics. *J. Electron. Mater.* **2019**, *48*, 1652–1659, https://doi.org/10.1007/s11664-018-06888-8.
10. Xiao, M.; Sun, H.; Zhou, Z.; Zhang, P. Bond Ionicity, Lattice Energy, Bond Energy, and Microwave Dielectric Properties of $Ca_{1-x}Sr_xWO_4$ Ceramics. *Ceram. Int.* **2018**, *44*, 20686–20691, https://doi.org/10.1016/j.ceramint.2018.08.062.
11. Sanderson, R. Multiple and Single Bond Energies in Inorganic Molecules. *J. Inorg. Nucl. Chem.* **1968**, *30*, 375–393, https://doi.org/10.1016/0022-1902(68)80464-6.
12. Luo, Y.-R. *Comprehensive Handbook of Chemical Bond Energies*; CRC Press: Boca Raton, FL, USA, 2007. https://doi.org/10.1201/9781420007282.
